# Circular RNAs in cell cycle regulation: Mechanisms to clinical significance

**DOI:** 10.1111/cpr.13143

**Published:** 2021-10-20

**Authors:** Wei Xiao, Juan Li, June Hu, Lingzhi Wang, Jiang‐Rong Huang, Gautam Sethi, Zhaowu Ma

**Affiliations:** ^1^ Health Science Center Yangtze University Jingzhou China; ^2^ Key Laboratory of Environmental Health Ministry of Education Department of Toxicology School of Public Health Tongji Medical College Huazhong University of Science and Technology Wuhan China; ^3^ The Second School of Clinical Medicine Yangtze University Jingzhou China; ^4^ Department of Pharmacology Yong Loo Lin School of Medicine National University of Singapore Singapore Singapore; ^5^ Cancer Science Institute of Singapore National University of Singapore Singapore Singapore

## Abstract

Circular RNAs (circRNAs), a type of non‐coding RNA, are single‐stranded circularized molecules characterized by high abundance, evolutionary conservation and cell development‐ and tissue‐specific expression. A large body of studies has found that circRNAs exert a wide variety of functions in diverse biological processes, including cell cycle. The cell cycle is controlled by the coordinated activation and deactivation of cell cycle regulators. CircRNAs exert mutifunctional roles by regulating gene expression via various mechanisms. However, the functional relevance of circRNAs and cell cycle regulation largely remains to be elucidated. Herein, we briefly describe the biogenesis and mechanistic models of circRNAs and summarize their functions and mechanisms in the regulation of critical cell cycle modulators, including cyclins, cyclin‐dependent kinases and cyclin‐dependent kinase inhibitors. Moreover, we highlight the participation of circRNAs in cell cycle‐related signalling pathways and the clinical value of circRNAs as promising biomarkers or therapeutic targets in diseases related to cell cycle disorder.

## Graphical abstract

CircRNAs exert a wide variety of functions in diverse biological processes, including cell cycle. The cell cycle is controlled by the coordinated activation and deactivation of cell cycle regulators, including cyclins, cyclin‐dependent kinases and cyclin‐dependent kinase inhibitors. This review summarizes the functions and mechanisms of circRNAs in cell cycle regulation and highlights the clinical value of circRNAs as promising biomarkers or therapeutic targets.

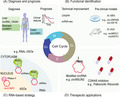

## 1 INTRODUCTION

Different RNA species, such as protein‐coding messenger RNAs (mRNAs) and non‐coding RNAs (ncRNAs), including circular RNAs (circRNAs), pseudogenes and long non‐coding RNAs (lncRNAs), display complex crosstalk. These RNA transcripts function as natural microRNA (miRNA) sponges or competing endogenous RNAs (ceRNAs) that communicate with and co‐regulate each other by competing or interacting with shared miRNAs. Deeper understanding of this novel RNA crosstalk will help shed light on gene regulatory networks and broaden the horizon on the study of circRNAs in human development and disease.^1^ CircRNAs are a covalently closed loop molecules without 5′ caps and 3′ poly (A) tails that confers resistance to RNase R, making them exceptionally more stable than linear RNAs.^2^ CircRNAs are highly enriched in eukaryotes, and several of these are evolutionary conserved and expressed in a cell type‐, developmental stage‐ and tissue‐specific pattern.^3^ Emerging studies have shed new light on how circRNAs execute diverse biological roles with cell type‐ or tissue‐specific expression in various organisms.^4^ Recently, independent studies have shown that the dysregulation of circRNAs is involved in a series of phenotypic changes, including proliferation and apoptosis.^4^, ^5^

The mammalian cell cycle is precisely regulated by diverse cyclins and cyclin‐dependent kinases (CDKs) as well as RB and p53‐related pathways.^6^, ^7^ Recently, several circRNAs have been implicated in the modulation of critical cell cycle regulators, including p53, pRB, CDKs, CDK inhibitors (CKIs) and cyclins. Therefore, dysregulation of the cell cycle‐associated circRNAs may be involved in pathophysiological process, including disease aetiology, and may serve as potential novel molecular targets for diagnosis, prognosis and treatment of diseases such as cancer.^8^, ^9^ However, the detailed functions and mechanisms of cell cycle regulation by circRNAs remain largely obscure. Recent studies, however, have revealed the diverse roles of miRNA and lncRNAs in cell cycle regulation.^10^, ^11^ To date, crosstalk between circRNAs and cell cycle has not been systematically elucidated. This review summarizes current research on the emerging landscape of circRNAs in the cell cycle regulation. We highlight the multifaceted functions of circRNAs and their underlying molecular mechanisms in cell cycle. A deeper understanding of circRNA functions in cell cycle may provide promising candidates for diagnosis and treatment of cell proliferation‐related diseases.

## 2 CIRCRNAS AS NOVEL REGULATORS OF CELL CYCLE

### 2.1 Characteristics of circRNAs

The widespread use of sequencing technologies and bioinformatics has facilitated the detection of circRNAs in diverse species, including humans, fungi, plants and other organisms. Emerging evidence demonstrates that circRNAs are widely expressed in different cells and tissues.^12^ Based on the diversity of source sequences, circRNAs are grouped into four major categories: exonic circRNAs (ecircRNAs), intronic circRNAs (ciRNAs), exonic–intronic circRNAs (EIciRNAs) and circRNAs generated from tRNAs (tricRNAs).^13^, ^14^

**FIGURE 1:**
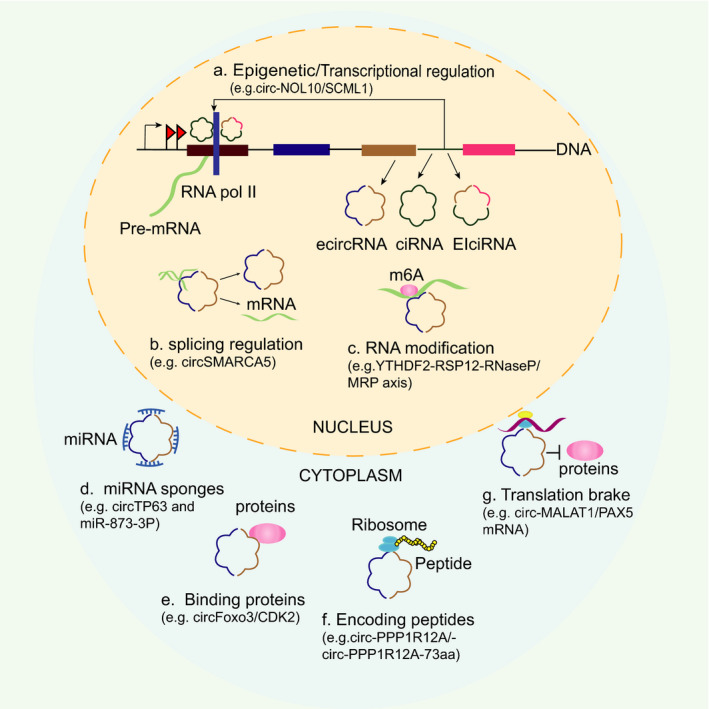
The basic molecular mechanisms of circRNAs. A diverse range of mechanisms have been described for circRNA regulation, including (a) epigenetic/transcriptional regulation, (b) splicing regulation, (c) RNA modification, (d) miRNA sponges, (e) binding proteins, (f) encoding peptides and (g) translation brake

Currently, different circRNAs are being constantly discovered and recognized as hotspots in diverse diseases, such as cancer and Alzheimer's disease.^15^ Accumulating evidence indicates that circRNAs manipulate different physiological and pathological processes via a diverse range of mechanisms, including transcription, miRNA sponge, protein sponge/decoy and translation.^5^ (Figure 1). Although circRNAs are generated in the nucleus, most are predominantly located in the cytoplasm,^16^, ^17^ suggesting specific rules for circRNA transportation or localization.^18^ On the one hand, most of circRNAs are localized in the cytoplasm, suggesting their roles in transcriptional and post‐transcriptional regulation; for example, certain circRNAs orchestrate gene expression via diverse mechanisms of action, including modulation of transcription, pre‐mRNA splicing and mRNA stability. On the other hand, circRNAs interact with a broad repertoire of biomacromolecules, thereby contributing to RNA or protein modifications, influencing functional peptide encoding. Furthermore, circRNAs exhibit developmental stage‐ and tissue‐specific expression.^19^ Moreover, unlike linear RNAs, circRNAs with loop structure are much more stable and resistant to RNases. Therefore, circRNA‐mediated gene regulation is a complex biological process that exists in a series of diseases including cancers, which provides avenues for prospective therapeutic interventions.

### 2.2 CircRNAs as mutifunctional players in cell cycle

CircRNAs play a diverse range of roles in multiple phenotypic aspects, including cell proliferation, apoptosis, invasion and metastasis.^20^ Therefore, it is worthwhile to further explore the interactive mechanisms between circRNAs and diseases. The elucidation of circRNA functions as effective molecular biomarkers or potential therapeutic targets will provide promising prospects for application, including early diseases diagnosis, treatment evaluation, prognosis prediction and even disease‐specific gene treatment.^21^ Clinically, circRNAs are differentially expressed in several of diseases, including cancers, indicating their regulatory role.^22^ CircRNAs have emerged as important modulators in a wide spectrum of pathophysiological processes as well as cell cycle regulation and proliferation.^23^, ^24^ Cyclins, CDKs and CKIs reportedly orchestrate diverse cellular processes involved in cell cycle regulation. The kinase activity of CDK/cyclin complexes is closely modulated by interplay between a serine/threonine‐specific catalytic core and regulatory subunits, termed as cyclins, which control phase transitions during the cell cycle. Under adverse conditions, CKIs act as brakes to impede cell cycle progression.^25^, ^26^, ^27^ The regulation of cell proliferation is a complex process driven by a large number of signals that lead to cell division and growth.^28^, ^29^, ^30^, ^31^ Thus, disorders affecting cell cycle regulation play an important role in excessive cell proliferation.^27^ Recent studies revealed that dysregulation of circRNAs exert accelerative and suppressive roles in human diseases, including cancer.

**FIGURE 2:**
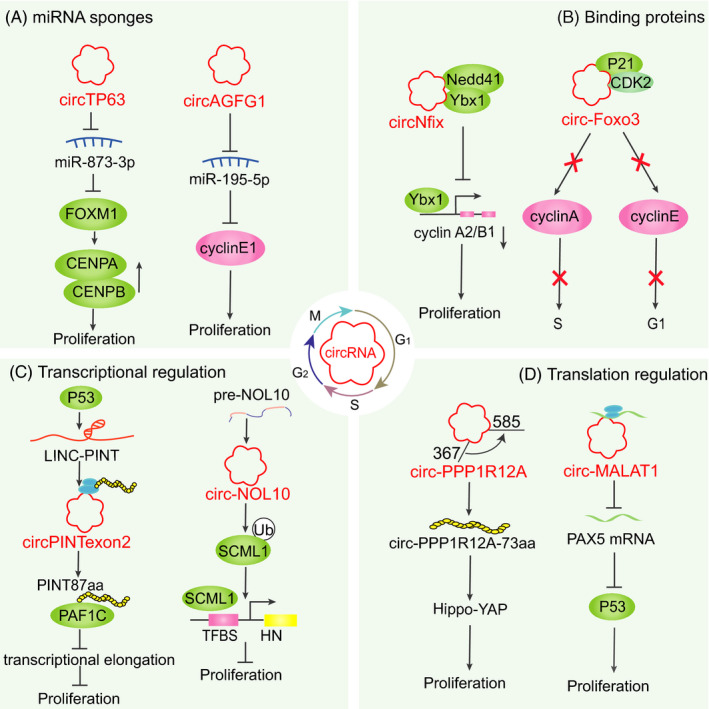
Mechanisms of action of circRNAs in cell cycle and proliferation. (A) CircRNAs act as molecular sponges to sequester miRNAs. (B) CircRNAs directly interact with RNA‐binding proteins (RBPs). (C) CircRNAs epigenetically mediate transcriptional regulation. (D) CircRNAs mediate translation regulation

Herein, we summarize the multiple regulatory functions of circRNAs in cell proliferation and apoptosis via diverse mechanisms (Figure 2). (i) *miRNA sponges*: Different sponge types can act as competing endogenous RNAs (ceRNA) to sequester miRNAs and dampen the gene expression of miRNA targets.^1^ Interestingly, circRNAs, such as circTP63 and circAGFG1, exert both promotive and suppressive roles to regulate the cell cycle by sponging miRNAs (e.g. miR‐873‐3p, miR‐195‐5p).^9^, ^32^ (ii) *Binding proteins*: CircRNAs affect cell cycle progression by binding to RNA‐binding proteins (RBPs), thereby affecting RNA processing and mRNA stability.^33^, ^34^ For example, ectopic expression of circ‐Foxo3 suppresses cell cycle progression by interacting with the cell cycle proteins CDK2 and p21, forming a ternary complex to block cell cycle progression – G_1_/S transition. CDK2 binds to cyclin A and cyclin E to promote cell cycle entry.^35^ Another important role of circRNAs is their influence on protein interactions. For example, circNfix enhanced the interaction between Y‐box binding protein 1 (Ybx1) and Nedd4l (an E3 ubiquitin ligase), thereby inhibiting the expression of cyclin A2 and cyclin B1 by inducing ubiquitination‐mediated Ybx1 degradation.^36^ (iii) *Transcriptional regulation*: CircRNAs function as epigenetic and transcriptional regulators to regulate gene expression. For example, circNOL10 facilitates transcription factor sex comb on midleg‐like 1 (SCML1) expression by suppressing its ubiquitination, thereby influencing the modulation of the humanin polypeptide family and suppressing cell cycle progression.^37^ An 87 amino acid peptide encoded by the circular form of the long intergenic non‐protein‐coding RNA p53‐induced transcript (LINC‐PINT) represses glioblastoma cell proliferation. This peptide suppresses the transcriptional elongation of multiple oncogenes by directly binding to polymerase‐associated factor complex (PAF1c). The expression of this peptide and its corresponding circRNA, circPINTexon2, was reportedly reduced in glioblastoma compared to normal tissues.^38^ Recent studies revealed that circRNAs might indirectly regulated gene transcription to induce cell proliferation (e.g. circERBB2 and circMRPS35).^8^, ^39^ (iv) *Translation regulation*: Recent studies uncovered that circRNAs act as mRNA translation brake and protein scaffolds to regulate gene expression. Circ‐MALAT1 serves as both microRNA sponge and mRNA translation brake to facilitate self‐renewal of hepatocellular cancer stem cells.^40^ CircPPP1R12A encodes a functional protein, circPPP1R12A‐73aa, which activates Hippo‐YAP signalling pathway to facilitate the growth and metastasis of colon cancer. Moreover, Peptide 17 (YAP‐specific inhibitor) markedly mitigated the promotive effect of circPPP1R12A‐73aa on colon cancer cells.^41^

Together, these reports indicate that circRNAs play a diverse range of roles for regulating cell cycle progression by different molecular mechanisms (Table 1). Therefore, understanding the mechanisms of circRNA‐mediated cell cycle may provide potential novel and promising therapeutic options suitable for clinical application.

**TABLE 1:** The emerging roles of circRNAs in cell cycle

CircRNA	Expression	Target	Functions and mechanisms	Disease	Refs
**CircRNAs during G_1_ and S phases**					
CircMYLK	Up	Cyclin D1	Increase the expression of cyclin D1 by sponging miR‐195	Laryngeal squamous cell carcinoma	^48^
CircPUM1	Up	Cyclin D1	Promote the expression of cyclin D1 by sponging miR‐326	Lung adenocarcinoma	^50^
CircNR3C1	Down	Cyclin D1	Suppress the expression of cyclin D1 by sponging miR‐27a‐3p	Bladder cancer	^56^
CircCHFR	Up	Cyclin D1	Promote the expression of cyclin D1 by sponging miR‐370	Vascular smooth muscle cells	^47^
CircAGFG1	Up	Cyclin E1	Up‐regulate the expression of cyclin E1 by sponging miR‐195‐5p	Triple‐negative breast cancer	^9^
Hsa_circ_0078710	Up	Cyclin A	Promote cell proliferation, migration, invasion and tumour growth by inducing the cell cycle progression	Hepatocellular carcinoma	^65^
Circ‐DB	Up	Cyclin A2	Promotes HCC growth and reduces DNA damage via the suppression of miR‐34a and the activation of deubiquitination‐related USP7	Hepatocellular carcinoma	^66^
CircNfix	Down	Cyclin A2 Cyclin B1	Ybx1 and Nedd4l suppress the expression of cyclin A2 and cyclin B1(S/M)	Cardiac regeneration	^36^
CircRNAs_100290	Up	CDK6	Promote the expression of CDK6 by sponging up miR‐29b family members	Oral squamous cell carcinoma	^69^
CircTCF25	Up	CDK6	Increase the expression of CDK6 by sponging miR‐103a‐3p and miR‐107	Bladder carcinoma	^71^
Circ‐ZEB1.33	Up	CDK6	Promote the expression of CDK6 by sponging miR‐200a‐3p	Hepatocellular carcinoma	^70^
Circ‐ZKSCAN1	Down	p21	Up‐regulate the expression of p21 by sponging miR‐1178‐3p	Bladder cancer	^74^
CircMMP9	Up	CDK4	CDK4 and aurora kinase A were involved in circMMP9/miR‐124 axis‐induced GBM tumourigenesis	Glioblastoma multiforme	^67^
Circ‐ITCH	Down	p21	Up‐regulate the expression of miR‐17 and miR‐224 target gene p21	Bladder cancer	^75^
BCRC‐3	Down	p27	Suppress cell proliferation by miR‐182‐5p/p27 axis	Bladder cancer	^76^
Circ‐LARP4	Down	p21	Activate the cell downstream p53/p21 by sponging miR‐761	Hepatocellular carcinoma	^78^
Circ‐0021977	Down	p21	MiR‐10b‐5p was shown to be a target of circ_0021977, and p21 and p53 are suggested to be putative target genes of miR‐10b‐5p	Colorectal cancer	^80^
CircYAP1	Up	p27	Suppress GC cells by targeting the miR‐367‐5p/p27 Kip1 axis	Gastric cancer	^81^
Circ‐Foxo3	Down	CDK2 p21	circ‐Foxo3–p21–CDK2 ternary complex arrested cell cycle progression	/	^35^
CircMTO1	Down	p21	Promote the expression of p21 by sponging miR‐9	Hepatocellular carcinoma	^77^
Circ‐FBXW7	Down	/	Up‐regulate FBXW7‐185aa in cancer cells inhibited proliferation and cell cycle acceleration	Brain cancer	^49^
CircPINTexon2	Down	/	The peptide directly interacts with polymerase‐associated factor complex (PAF1c) and inhibits the transcriptional elongation of multiple oncogenes	Glioblastoma	^38^
**CircRNAs during G_2_ and M phases**					
hsa_circ_0136666	Up	CDK6	Promote breast cancer progression by sponging miR‐1299 and targeting CDK6	Breast cancer	^55^
hsa_circ_0035483	Up	Cyclin B1	Promotes autophagy and tumour growth and enhances gemcitabine resistance in CRC by regulating hsa‐miR‐335/CCNB1	Colorectal cancer	^54^
CircNOL10	Down	/	Promote the apoptosis of lung cancer cells by regulating the humanin polypeptide family and affect multiple signalling pathways	Lung cancer	^37^

## 3 CIRCRNA FUNCTIONS IN CELL CYCLE

**FIGURE 3:**
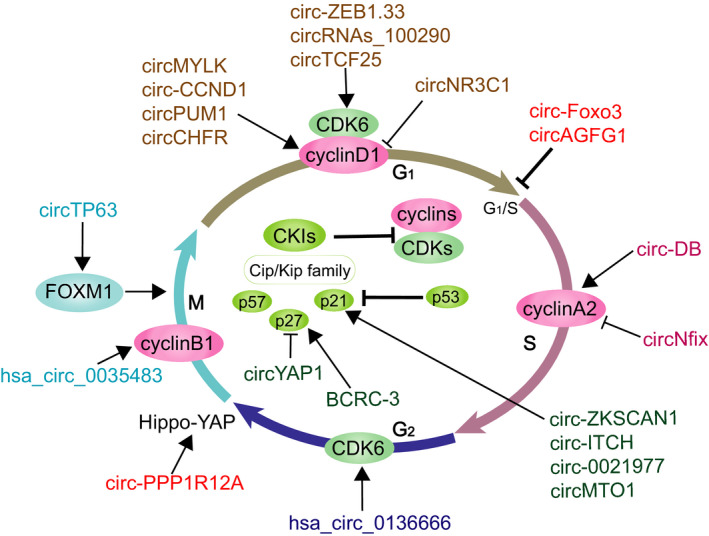
CircRNA impact cell cycle progression by regulating diverse cell cycle regulators. Several circRNAs control the expression of cyclins/CDKs, CKIs and participate in cell cycle regulation. M, mitotic phase; S, synthesis phase; G, gap phase; G_1_, the gap after cell division and before S phase is called the G_1_ phase; G_2_, the gap after S phase and before the next cell division is called the G_2_ phase

CircRNAs can specifically interact with different partner molecules, including DNA, RNA and proteins, and function as key regulators for mediating a diverse range of phenotypic changes. In this section, we focus on the expanding landscape of circRNAs in cell cycle regulation, including regulation of cyclins, CDKs, CKIs and related pathways (Table 1, Figure 3).

### 3.1 CircRNAs regulate cyclins and CDKs

Cyclins and CDKs play versatile roles as regulators in G_1_/S phase and G_2_/M phase transitions. The current classification of cyclins and CDKs is based on functional relevance and evolutionary conservation.^42^, ^43^ In the ‘classical’ model of the mammalian cell cycle, specific CDK–cyclin complexes drive the numerous events that occur during the interphase in a sequential and orderly manner.^27^ At the G_1_/S transition point, cyclin E–CDK2 promotes G_1_/S progression by phosphorylating pRB as well as several DNA replication‐related proteins.^44^ CDK1 and cyclin B mediate the transition from G_2_ into M phase. Although cyclin B (with subtypes B1 and B2) is expressed in G_1_, S and G_2_ phases, its peak expression and function are found in the M phase.^45^, ^46^ A plethora of cell cycle constituents manipulate the machinery either directly or are targeted by ncRNAs, including circRNAs. Interestingly, they can perform a few of these tasks alone, without the need for CDK/cyclin complex formation or kinase activity.^25^

Current studies are focused on the effect of circRNAs on tumour progression via regulation of diverse cyclins/CDKs and their downstream genes. Mounting evidence indicates that circRNAs function as molecular sponges that absorb miRNAs to regulate cell or tumour progression. Numerous lines of evidence have proven that miRNAs may serve as pivotal modulators of the cell cycle, including G_1_ to S transition. CircRNAs reportedly promote the cell cycle transition by interacting with cyclin D1 and corresponding regulators. For example, Yang *et al*. identified abnormal overexpression of circRNA circCHFR in the ox‐LDL‐mediated vascular smooth muscle cells (VSMCs); circ‐CHFR knockdown inhibited VSMC proliferation and migration via circCHFR/miR‐370/FOXO1/CyclinD1 axis, implicating circRNAs in VSMCs and atherosclerosis.^47^ Duan *et al*. found that circMYLK was markedly up‐regulated in laryngeal squamous cell carcinoma (LSCC). Gain‐of‐function experiments indicated that circMYLK facilitated LSCC cell proliferation and G_1_/S cell cycle transition. Thus, circMYLK elevated cyclin D1 expression by sponging miR‐195.^48^ Furthermore, CCND1‐derived circ‐CCND1 as a crucial player that was significantly elevated in LSCC and was associated with aggressive clinical features and adverse prognosis. Loss‐of‐function studies uncovered that circ‐CCND1 suppressed LSCC cell proliferation and tumour growth. Mechanically, circ‐CCND1 interacts with HuR protein to strengthen CCND1 mRNA stability as well as promotes CCND1 translation by sponging miR‐646. This study identified that circ‐CCND1 post‐transcriptionally promotes LSCC tumourigenesis.^126^ Another study identified circPUM1 was significantly up‐regulated in lung adenocarcinoma and increased cyclin D1 and Bcl‐2 expression by sponging miR‐138‐5p, thereby facilitating cell proliferation, migration and invasion of lung adenocarcinoma.^50^ Li *et al*. identified high expression of hsa_circ_000984 in non–small cell lung cancer (NSCLC) tissues and cell lines, which was linked to advanced TNM stage and lymph node metastasis. Functional assays demonstrated that hsa_circ_000984 silencing inhibited NSCLC cell proliferation, migration, invasion and EMT. In addition, hsa_circ_000984 exerts oncogenic effects by regulating the activation of Wnt/β‐catenin pathway, as determined by detecting its influence on the expression levels of β‐catenin, c‐myc and cyclin D1.^51^ Thus, the circ‐CMPK1/miR‐302e/cyclin D1 signalling pathway plays an important regulatory function in NSCLC, and targeting this axis may be an effective treatment strategy.^52^ Another circRNA, circ5615, was significantly up‐regulated in colorectal cancer (CRC) and exhibited an oncogenic role via impacting the cell cycle. Silencing circ5615 suppressed CRC proliferation via miR‐149‐5p sponging, leading to increase in expression of tankyrase, a regulator of β‐catenin stabilization, thereby inducing β‐catenin and cyclin D1 expression.^53^ hsa_circ_0035483 promotes gemcitabine‐induced autophagy and augments gemcitabine resistance in renal cancer cells. Silencing hsa_circ_0035483 increases gemcitabine sensitivity and inhibits tumour growth via regulation of miR‐335/CCNB1 axis.^54^ hsa_circ_0136666 promotes breast cancer cell proliferation and induces G_2_/M phase transition by sponging miR‐1299 and up‐regulating CDK6 expression.^55^ CircRNAs regulate cell cycle regulators such as cyclins, CDKs, CKIs, crucial cell cycle effectors. These reports suggest that several circRNAs promote cell cycle transition, thereby playing oncogenic roles.

On the other hand, several circRNAs inhibit the cell cycle transition via diverse molecular mechanisms. For example, circNR3C1 significantly suppresses cell proliferation and cell cycle progression by sponging miR‐27a‐3p, thereby inhibiting cyclin D1 expression; it was found to be significantly down‐regulated in bladder cancer.^56^ Another study found reduced levels of hsa_circ_0000096 in gastric cancer tissues and cell lines, and its correlation with invasion and TNM stage. Functional assays showed that hsa_circ_0000096 knockdown inhibited cell proliferation by arresting the cell in the G_0_/G_1_ phase. Moreover, hsa_circ_0000096 alters protein levels of CDK6, cyclin D1, MMP‐2 and MMP‐9.^57^ Taken together, these reports indicate that circRNAs regulate the G_1_/S transition by directly or indirectly affecting cyclins.

There are, of course, other ways to influence tumour progression, such as by modulating activation of transcription factors, affecting DNA damage or overcoming chemoresistance, or through yet unknown mechanisms.^58^, ^59^, ^60^, ^61^, ^62^ Triple‐negative breast cancer is a lethal disease.^63^, ^64^ Yang *et al*. found that high expression of circAGFG1 in triple‐negative breast cancer (TNBC) was linked to clinical stage, pathological grade and poor prognosis. Functional assays confirmed that circAGFG1 could promote TNBC cell proliferation and tumourigenesis *in vivo*. CircAGFG1 acts as an miRNA sponge; its binding to miR‐195‐5p increases cyclin E1 expression, indicating that circAGFG1 could serve as a diagnostic biomarker and therapeutic target in TNBC.^9^ Xie *et al*.^65^ proved that the elevated expression of has_circ_0078710 promoted cell proliferation, migration and tumour growth in hepatocellular carcinoma (HCC), which acts as a ceRNA for miR‐31 to increase HDAC and CDK2 expression and regulate expression of other cell cycle components, including CDK4, cyclin D1, cyclin A and p21. Zhang *et al*.^66^ reported that the adipocyte‐derived exocirc‐deubiquitination (exo‐circ‐DB) could sponge miR‐34a to activate the USP7/CyclinA2 signalling pathway, thus promoting HCC growth and metastasis and reducing DNA damage.

Accumulating evidence indicates that tumour cells require specific interphase CDKs for proliferation.^27^ Some of these CDKs are directly up‐ or down‐regulated by circRNAs, while others are indirectly involved. For example, Wang *et al*. identified circMMP9 up‐regulation and its oncogenic role in glioblastoma multiforme (GBM). They found that eukaryotic initiation factor 4A3 (eIF4A3) physically interacts with matrix metallopeptidase 9 (MMP9) mRNA transcript to induce its cyclization, promoting circMMP9 production. CircMMP9 enhanced GBM cell proliferation and tumourigenesis via CDK4 and aurora kinase A (AURKA) by sponging miR‐124.^67^ Similarly, circ_001621 facilitated cell proliferation and migration in advanced osteosarcoma via the miR‐578/CDK4/MMP9 axis, highlighting its role as an oncogene by activating VEGF‐dependent progression.^68^ In oral squamous cell carcinoma (OSCC), circRNAs_100290 was significantly up‐regulated and repressed CDK6 expression and cell proliferation by sponging miR‐29b.^69^ Circ‐ZEB1.33 was found to promote HCC proliferation by regulating CDK6 expression via competitively binding miR‐200a‐3p. Circ‐ZEB1.33 facilitated HCC proliferation by elevating the percentage of S phase mediated by CDK6/Rb. The identification of this key circ‐ZEB1.33/miR‐200a‐3p/CDK6/ axis provides new insight into the potential of circ‐ZEB1.33 as an indicator of prognosis in HCC patients.^70^ Zhong *et al*.^71^ screened differential circRNA expression profiles and identified that the circTCF25‐miR‐103a‐3p/miR‐107‐CDK6 axis facilitates proliferation and migration in bladder carcinoma, thus highlighting the crucial role of circTCF25 and its potential as prospective molecular markers in bladder cancer. cZNF532 regulates pericyte biology by sponging miR‐29a‐3p and inducing CDK2 up‐regulation in pericytes under diabetic stress. Silencing cZNF532 or overexpressing miR‐29a‐3p aggravated streptozotocin‐induced retinal pericyte degeneration and vascular dysfunction.^72^ Thus, circRNAs regulate cell cycle‐related genes in several diseases, suggesting their potential for therapeutic intervention in disease progression.

### 3.2 CircRNAs regulate CKIs

Considering that most cyclins facilitate CDK activity, CKIs repress CDK activity.^25^ Based on homology and CDK specificity, CKIs are subdivided into two families: Cip/Kip (CDK‐interacting/kinase‐inhibiting protein) family and INK4 CKI family. The Cip/Kip family comprises p57 (Cdkn1c), p27 (Cdkn1b) and p21 (Cdkn1a) that mainly inhibit CDK2 activity, thereby inducing cell cycle arrest. INK4 CKI family includes p15 (CDKN2B), p16 (CDKN2A), p18 and p19.^73^ Recent studies showed that circRNAs orchestrate cell cycle progression by mediating cell cycle inhibitors, such as p21 and p27, in cancers. p21 serves as a crucial regulator of p53‐induced cell cycle arrest during distinct checkpoints. p27/Kip1 modulates the cell cycle by repressing the checkpoint kinase CDK2/cyclin E and obstructing cell cycle progression via the G_1_/S transition.

Emerging studies suggest that circRNAs regulate cell proliferation and progression by affecting the circRNAs/miRNA/p21 axis. Bi *et al*. identified down‐regulation of circ‐ZKSCAN1 in bladder cancer tissues and cell lines; circ‐ZKSCAN1 suppresses cell proliferation by sponging miR‐1178‐3p to increase p21 expression.^74^ Similarly, circ‐ITCH that acts as a tumour suppressor to inhibit cell proliferation and tumourigenesis was also down‐regulated in bladder cancer. Circ‐ITCH promotes the expression of miRNA target gene p21 and PTEN by competitively binding miR‐17 and miR‐224.^75^ Recent studies indicate that p27 is also regulated by circRNAs. CircBCRC‐3 inhibits bladder cancer proliferation through miR‐182‐5p sponging to induce p27 expression. Furthermore, methyl jasmonate significantly elevated the expression of BCRC‐3, leading to a marked up‐regulation of p27.^76^ CircRNAs impact cell cycle control in other cancer types as well. For example, reduced circMTO1 expression could serve as a prognosis predictor and poor survival in HCC patients. Silencing circMTO1 could reduce the levels of p21, an oncogenic miR‐9 target, thereby facilitating HCC cell proliferation and invasion.^77^ Another loss of function study showed that circLARP4 inhibits HCC cell proliferation and regulates cell cycle arrest. Mechanistic studies revealed that circLARP4 binds to miR‐761 to activate downstream p53/p21 targets and promote RUNX3 expression, thereby regulating disease progression.^78^ Overexpression of circPCNX specifically interfered with the binding between AUF1 and p21 (CDKN1A) mRNA, promoting p21 mRNA stability and elevating its production. Silencing circPCNX increased AUF1–p21 mRNA binding, thus reducing p21 production and promoting cell division.^79^

Furthermore, circRNAs regulate CKIs through other mechanisms. CircRNAs target miRNAs that in turn target tumour suppressor genes. This axis is used to regulate cell cycle progression and considered an indicator of healing. For example, circ_0021977 was found to suppress CRC proliferation, migration and invasion by modulating the miR‐10b‐5p/p21 and p53 axis. Low circ_0021977 expression in CRC patients was correlated with higher TNM stage and poorer prognosis.^80^ Additionally, circYAP1 acts as a tumour suppressor to restrain cell growth and invasion via targeting the miR‐367‐5p/p27 Kip1 axis, which may offer a promising prognostic indicator of survival in patients with gastric cancer.^81^

### 3.3 CircRNAs mediate other pathways

In addition to the p53 pathway, many other signalling pathways are regulated by a range of circRNAs in the cell cycle. Here, we discuss two circRNA‐mediated signalling pathways involved in cell cycle regulation: RB‐E2F pathway and PI3K/AKT pathway.

#### 3.3.1 RB‐E2F Pathway

The transition from G_1_ to S phase is regulated by the interplay between CDKs and retinoblastoma protein (Rb) phosphorylation, thereby releasing E2F transcription factors to facilitate the expression of S phase genes. It is widely recognized that pRB targets members of the E2F family.^82^ As subgroups of E2F family, E2F1, E2F2 and E2F3 are the ‘activating’ E2Fs that function as transcriptional activators to induce the G_1_/S transition.^83^, ^84^ The pRB‐E2F pathway plays significant roles in manipulating the mammalian cell cycle progression. Moreover, emerging studies report the participation of dysregulated circRNAs in cell cycle progression via maintenance of proliferative signalling in a plethora of diseases. For example, circCAMSAP1 (hsa_circ_0001900) was dramatically increased in CRC tissues and facilitated CRC malignant behaviour. Mechanistical analysis indicated that circCAMSAP1 cyclization was regulated by epithelial‐splicing regulatory protein 1 (splicing factor). Moreover, circCAMSAP1 functioned as an miR‐328‐5p sponge and up‐regulated E2F1 expression in CRC.^85^ Cen *et al*. reported aberrant circSDHC expression profile in renal cell carcinoma (RCC) patients; it promoted RCC proliferation and invasion by competitively binding miR‐127‐3p to up‐regulate the downstream gene, CDKN3 and the E2F1 pathway.^86^ Similarly, circ‐Foxo3 was highly expressed in ageing cardiac tissues and facilitated cellular senescence by interacting with the anti‐senescence proteins E2F1 and ID1 to influence impacted their subcellular translocation. Moreover, overexpression of E2F1 in miR‐205‐expressing cells could partly reverse the senescent phenotype.^87^ Another study reported that circRNA CDR1 promotes E2F3 expression by binding miR‐7‐5p, thereby facilitating nasopharyngeal carcinoma growth and glucose metabolism.^88^ Highly expressed hsa_circ_0008039 exerted oncogenic effects in breast cancer, while its depletion markedly inhibited cell cycle progression and proliferation via sponging miR‐432‐5p and elevating E2F3 expression.^89^

#### 3.3.2 PI3K/AKT Pathway

The phosphoinositide 3‐kinase (PI3K) pathway plays an integral role in many cellular processes and is frequently altered in cancer, contributing to tumour growth and survival.^90^, ^91^ Small molecule inhibitors have been developed that target the three major nodes of this pathway: PI3K, AKT and mammalian target of rapamycin (mTOR).^92^, ^93^, ^94^, ^95^ CircRNAs have also been shown to target this axis. For example, a study identified that silencing of circ‐ZNF609 specifically blocks the G_1_/S transition via inhibiting phosphorylated Rb:Rb ratio. By contrast, circ‐ZNF609 expression promotes cell proliferation in rhabdomyosarcoma, highlighting circ‐ZNF609 as a new regulator of cell proliferation‐related pathways that abrogates p‐Akt proteasome‐dependent degradation.^96^ Similarly, circ‐IGF1R was significantly up‐regulated, acting as an oncogene in HCC. siRNA‐mediated knockdown of circ‐IGF1R inhibited cell proliferation and triggered cell apoptosis. Further analysis indicated that circ‐IGF1R activates PI3K/AKT signalling pathway, contributing to cell cycle.^97^ Another study confirmed that exosomal circNRIP1 could be transported between gastric cancer cells and promoted the proliferation, migration and invasion by sponging miR‐149‐5p to activate the AKT1/mTOR signalling pathway.^98^ Despite being the most well‐studied signalling pathway implicated in cancer molecular mechanisms, the physiological functions of these kinases in cells and organisms are far more complex than previously assumed.^99^ Therefore, a comprehensive investigation of underlying mechanism may also provide novel insights into the crosstalk in PI3K/AKT pathway and cell cycle regulation.

## 4 POTENTIAL DIAGNOSTIC AND THERAPEUTIC APPLICATIONS OF CIRCRNAS

**FIGURE 4:**
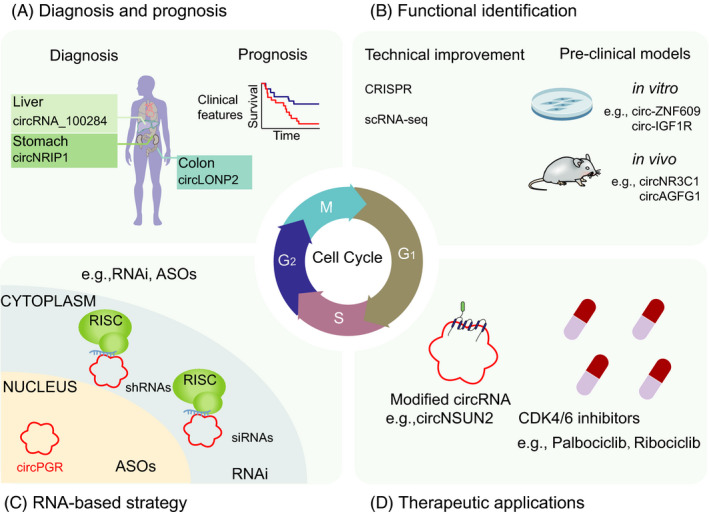
The potential clinical application of circRNAs. (A) CircRNAs can be detected in tumour biopsies of patients, and are potential diagnostic and prognostic biomarkers. (B) The functional identification of circRNAs has been elucidated in pre‐clinical models. (C) RNA‐based strategy can effectively target circRNAs in the cytoplasm and nucleus. (D) Combination of circRNA targeting and conventional CKIs enhances therapeutic efficacy through diverse delivery tools *in vivo*

Emerging studies have shown that certain circRNAs act as promising biomarkers and therapeutic targets in various diseases. The expression patterns and characteristics of circRNAs make them perfect candidates as valuable biomarkers to improve diagnostic efficacy. Furthermore, circRNAs are abundant in human body fluids, such as blood and saliva, which makes them easily accessible and easy to detect, and thus, viable biomarkers for cancer detection, particularly via liquid biopsies. Moreover, a series of circRNAs, such as F‐circEA, are more suitable for developing biomarkers than the linear RNA transcripts.^100^, ^101^ Thus, circRNAs may function to be prospective diagnostic biomarkers or novel therapeutic targets for disease treatment. In the following sections, we discuss circRNAs as prospective tools in the clinic for diagnosis and treatment of different disease types (Figure 4).

### 4.1 CircRNAs as promising biomarkers

Considering that circRNAs exert key roles in disease progression and cell cycle as well as feature abundance, conservation stability and prevalence,^102^, ^103^, ^104^ they are appropriate candidates for biomarkers. High‐throughput sequencing has identified a number of circRNAs that may be prospective non‐invasive biomarkers for diagnosis and prognosis of distinct diseases. For instance, circRNA_100284 accelerates the cell cycle and promotes cell proliferation in the malignant transformation of human hepatic cells.^19^ However, there are only few studies on circulating circRNAs as tumour biomarkers. As tumour‐derived exosomes contain tumour‐specific circRNAs, these may be used as tumour biomarkers. A recent study confirmed that exosomal circNRIP1 could be transported between gastric cancer cells and promoted proliferation, migration and invasion by activating the AKT1/mTOR signalling pathway.^98^ Similarly, other studies confirmed cancer cell‐specific expression of circRNAs, such as circTCF25 and circRNAs_100338.^71^, ^105^ Thus, combining specific circRNAs with other biomarkers could improve the accuracy and specificity of diagnosis for a diverse range of diseases. The clinical applications of circRNAs continues to expand, with their use in a wide range of patient samples, including circ_0051443 and circFOXO3.^106^, ^107^ Taken together, the combination of circRNAs and conventional biomarkers will improve diagnostic specificity and sensitivity in specific diseases or cell subtypes.

### 4.2 CircRNAs as therapeutic targets

Several studies have focused on the clinical utility of circRNAs as promising therapeutic targets in many diseases.^54^, ^108^ Recent studies demonstrated that the CRISPR‐Cas13 technique can be used to knockdown circRNAs without influencing the homologous mRNAs in mouse embryos.^109^, ^110^ Moreover, single‐cell RNA sequencing (scRNA‐seq) provides a novel perspective for exploring the functions of circRNA at single‐cell resolution to understand their cell‐specific regulation as well as cell cycle regulation.^111^, ^112^ A recent study reported a new data analysis technique for circRNA – CIRI‐long – that uses nanopore long reads and enables unbiased reconstruction of full‐length circRNA sequences.^113^ CircRNAs exert proper biological functions through dynamically regulated biogenesis and degradation; however, the mechanism of their degradation remains largely unknown.^114^ A coordinated biogenesis and decay of circRNAs is essential in eukaryotic cells. Recently, Fischer *et al*.^115^ reported a novel structure‐derived circRNA degradation mechanism that selectively decayed highly structured RNAs via UPF1 and G3BP1. Although a few reports indicate the involvement of some endonucleases in circRNA cleavage under certain circumstances, the detailed underlying molecular mechanisms remain unclear. Therefore, the general degradation pathway for circRNAs remains abstruse.^114^

An understanding of gene overexpression or knockdown mechanisms may provide new clues for the therapeutic targeting of circRNAs. Currently, RNA‐based therapeutic approaches mainly include RNA interference (RNAi) and antisense oligonucleotides (ASOs), which can be designed to target a large and heterogeneous class of transcripts.^116^ For promotive circRNAs, specific shRNAs or siRNAs targeting the back‐splice junction, which can avert interference by homologous linear mRNA expression, were utilized to achieve circRNA‐specific knockdown.^117^ ASOs have shown promise in targeting ncRNAs in patients with distinct tumours. For example, targeting circPGR by ASO reduced breast cancer cell growth by regulating cell cycle genes; thus, circPGR may be a novel and promising therapeutic target for oestrogen receptor‐positive breast cancer.^118^ Conversely, for suppressive circRNAs, the overexpression vectors fostering back‐splicing consisted of flanking introns with reverse complementary sequences and circRNA‐forming exons, thereby influencing cell cycle progression.^19^ Optimized RNA‐targeting CRISPR/Cas13d technology outperforms shRNA in identifying functional circRNAs.^119^ To date, prominent advances in many cancer models could pave the way for their utilization in cell cycle regulation.

Currently, several specific CDK4/6 inhibitors targeting CDK‐Rb‐E2F pathway are either approved or in clinical trials for the treatment of different cancer types.^120^ For example, a clinical phase II trial reported that the combination of CDK4/6 inhibitors and conventional chemotherapy (carboplatin and gemcitabine) increases progression‐free survival in TNBC patients.^121^ Similarly, a combination of palbociclib and letrozole to target CDK4/6 was effective in metastatic HER2‐negative ER‐positive breast cancer in post‐menopausal women.^122^ Ribociclib, the pyridopyrimidine palbociclib, and its isosteres ribociclib and abemaciclib are highly selective for CDK4 and CDK6, and induce G_1_/S arrest exclusively.^123^ However, clinical applications of targeting circRNAs is still in its infancy, extensive studies need urgently to develop progressive techniques and efficient agents for clinical applications. Therefore, the combination of CKIs with specific circRNAs is a promising treatment strategy for several diseases. Moreover, advances in cancer models *in vivo* can pave the way for their utilization in cell cycle‐related diseases. However, more clinical trials are needed to drive the development of ncRNA‐based diagnostic tests and therapeutic interventions for cancer patients.

## 5 CONCLUSION AND FUTURE PRESPECTIVES

In this review, we emphasized the expanding functions of circRNAs in cell cycle regulation and their potential diagnostic and therapeutic application in diverse diseases. Significantly, accumulating evidence indicates that circRNAs regulate cell cycle by directly or indirectly interacting with cyclins/CDKs. Moreover, circRNAs orchestrate cell cycle progression via distinct molecular mechanisms by interacting with RNA, DNA or proteins. The functions and mechanisms of circRNAs in cell cycle regulation are largely focused on the G_1/_S phase transition and involve miRNA sponging, protein interaction, transcription and translation. Currently, circRNAs have been recognized to have distinct expression profiles in different diseases and might exert multifunctional roles, making them promising biomarkers for diagnosis, prognosis and treatment.

Despite these recent advances, the exact mechanisms of action of circRNAs remain largely unknown, certain challenges and limitations in this field need to be overcome. Therefore, the following key questions should be answered: (i) As sequence conservation was identified in most cyclin/CDK families, are circRNAs that regulate these cyclins/CDKs also conserved? (ii) Considering that cyclins are periodic, are the circRNAs that regulate these cyclins also periodic? (iii) In light of the diversity of cyclin families and encoded peptides, is circRNA coding heterogeneous from the same source DNA, and what are the main factors and mechanisms underlying this dynamic regulation?

CircRNAs are dynamic as well as specific to tissue type, genetics and stage, suggesting their potential diagnostic and prognostic significance in multiple types of cancer. The immunomodulatory activity of CDK4/6 inhibitors focuses on their regulatory effects on the proliferation and activation of immune cells. CDK4/6 inhibition resulted in significant positive therapeutic effect in patients with advanced hormone‐positive breast cancer. However, specific mechanisms of resistance for tumour cell evasion remain largely unknown.^124^, ^125^ Therefore, the combination of specific circRNAs and CKIs could enhance the therapeutic efficacy in complex diseases. Moreover, future studies should focus on elucidating the precise mechanisms of action of circRNAs and facilitate the development of novel technologies and methods to identify important signalling pathways involved in cell cycle regulation.

## CONFLICT OF INTEREST

The authors declare no conflict of interest.

## AUTHOR CONTRIBUTIONS

W.X. and J.L. collected the related paper and drafted the manuscript. J.H. and L.W. helped to modify the manuscript and figures. Z.M. conceived the review article Z.M., G.S. and J.R.H designed the review and revised the manuscript. All authors read and approved the final manuscript.

## Data Availability

The data sharing is not applicable to this article.
